# Morphological and elemental mapping of gallstones using synchrotron microtomography and synchrotron X‐ray fluorescence spectroscopy

**DOI:** 10.1002/jgh3.12171

**Published:** 2019-03-29

**Authors:** Mohana Bakthavatchalam, Jayanthi Venkataraman, Ramya J Ramana, Mayank Jain, Balwant Singh, Arul K Thanigai, Vaithiswaran Velyoudam, Saravanan Manickam Neethirajan, Manoj K Tiwari, Ashish K Agarwal, Narayana S Kalkura

**Affiliations:** ^1^ Crystal Growth Centre Anna University Chennai India; ^2^ Institute of GI Sciences, Gleneagles Global Hospitals and Health City Chennai India; ^3^ Indus 2 Raja Ramanna Centre for Advance Technology Indore India; ^4^ Department of Physics, Energy and Biophotonics Lab AMET Chennai India

**Keywords:** cholesterol, elemental, gallstones, microtomography, pigment, synchroton

## Abstract

**Background and Aim:**

Regional differences in gallstone (GS) composition are well documented in the Indian subcontinent. The reasons for the same are unknown. Etiopathogenesis of GS remains elusive despite advances in instrumentation. This was an in‐depth analysis of the chemical, structural, and elemental composition of GS with special reference to synchroton studies.

**Methods:**

We used high‐end sensitive analytical complementary microscopic and spectroscopic methods techniques, such as X‐ray diffraction, scanning electron microscopy, Fourier transform infrared, synchrotron X‐ray fluorescence spectroscopy (SR‐XRF), and 2D and 3D synchrotron microtomography (SR‐μCT), to study the ultra structure and trace element composition of three major types of GS (cholesterol, mixed, and pigment). SR‐XRF quantified the trace elements in GS.

**Results:**

The cholesterol GS (monohydrate and anhydrate) were crystalline, with high calcium content. The pigment GS were amorphous, featureless, black, and fragile, with high calcium bilirubinate and carbonate salts. They had the highest concentration of iron (average 31.50 ppm) and copper (average 92.73 ppm), with bacterial inclusion. The mixed stones had features of both cholesterol and pigment GS with intermediate levels of copper (average 20.8 ppm) and iron (average 17.78 ppm).

**Conclusion:**

SR‐μCT has, for the first time, provided cross‐sectional computed imaging delineating the framework of GS and mineral distribution. It provided excellent mapping of cholesterol GS. SR‐XRF confirmed that pigment GS had high concentrations of copper and iron with bacterial inclusions, the latter possibly serving as a nidus to the formation of these stones.

## Introduction

Gallstone (GS) disease is a common problem in clinical practice. A majority of these stones is asymptomatic.^1^ These are classified as cholesterol, pigment, and mixed stones. In the Indian subcontinent, there are regional differences in the composition of GS. They are predominantly cholesterol or mixed in the northern, eastern, and western parts of the country and mixed and pigment in the southern states of the subcontinent.^2^, ^3^

In our previous study, we noted that the GS from south India, on proton‐induced X‐ray emission (PIXE) analysis,^4^ had high iron content, and this was attributed to the significant dietary consumption of *Tamarindus indica*.^5^ Recent published data from Hyderabad^6^ stated that pigment GS in south India was possibly due to raised serum bilirubin levels because of a possible variant in the UGT1A1 gene involved in the glucuronidation of free bilirubin. A study from Jharkhand, North India showed high prevalence of pigment GS, and this was attributed to high iron content in the soil.^7^ Thus, pigment GS formation in India appears to be due to interplay between environmental, host, and genetic factors and seems unrelated to infection or hemolysis.

Comprehensive knowledge of the structural features and chemical/elemental composition of GS is important in understanding the etiopathogenesis of its formation. Various complementary sophisticated analytical instrumentations, such as scanning electron microscopy/energy‐dispersive X‐ray analysis (SEM/EDAX), Fourier transform infrared (FTIR), Raman and electron paramagnetic resonance (EPR) spectroscopy, X‐ray diffraction (XRD), cathodoluminescence, thermal analysis, etc., have thrown some light on the composition and structural details of GS. We have, in the past, reported the biochemical, structural, and elemental composition of GS using some of these analytical techniques^2^, ^4^, ^8^, ^9^, ^10^, ^11^, ^12^, ^13^ and also characterized the composition of gallbladder bile.^14^ These studies were an attempt to study the key factors that initiate and promote the formation of GS, which even to this day remains elusive.

The aim of the present study was therefore to perform sequential morphological and multielemental analysis of 10 randomly selected representative GS from south India using highly sensitive complementary microscopy and spectroscopy techniques with the intent to obtain an insight into the gross morphological and elemental mapping of GS.

## Methods

A total of 45 GS were obtained from patients undergoing cholecystectomy at Gleneagles Global Health City, Chennai, Tamil Nadu after informed consent. These were washed with triple‐deionized water and dried at 37°C. Based on the gross morphological^9^ and biochemical composition^8^ (Table 1), 10 GS samples were segregated: cholesterol (CH): 6 and pigment (P) and mixed (M) 2 each.

**Table 1:** Characterization of cholesterol, mixed, and pigment gallstone

		Biochemistry	
	Morphology	Cholesterol	Bilirubinate salts
Cholesterol	White or yellow hard and facile (5–10 mm)	85–90%	10–15%
Mixed	Variegated color irregular surface, prominent nidus (8–15 mm)	Predominant cholesterol or bilirubinate salts	
Pigment	Black amorphous, irregular surface no nidus (6–8 mm)	10–15%	>90%

The representative GS were subjected to sequential evaluation by optical microscopy; XRD at Sri Ramaswamy Memorial University, Chennai, India; FTIR; SEM at Crystal Growth Centre, Anna University, Chennai, India; and synchrotron microtomography and X‐ray fluorescence spectroscopy (XRF) at Raja Ramanna Centre for Advance Technology Indore, India. Table 2 summarizes the principles and interpretation of the various analytical techniques. While most of the techniques mentioned above provide information on the morphological and chemical composition of GS, synchrotron micro tomography provided additional information on the cross‐sectional mineral distribution within the GS.

**Table 2:** Principles and interpretation of various analytical methods for cholesterol and pigment gallstones. (mixed gallstones [GS] is a combination of findings of cholesterol and pigment GS)

	Detection	Representation	Cholesterol anhydrous	Cholesterol monohydrate	Pigment
XRD (phase)	Crystalline	Peaks	Present	Present	
	Amorphous				Present
FTIR	Functional groups	Wavelength per cm	2800–2950		1000–1500
SEM	Surface	Micrograph images	Platy and needle like crystals		Noncrystalline
SR‐μCT (morphology)	2D gray	Gray pictures	Cross‐sectional crystalline appearance		Fragile
	3D color	Color pictures	Distribution of minerals		No minerals
SR‐XRF	Elemental composition in ppm	Peaks	Elements as per periodic table		

FTIR, Fourier transform infrared; SEM, scanning electron microscopy; SR‐μCT, synchrotron microtomography; SR‐XRF, X‐ray fluorescence spectroscopy; XRD, X‐ray diffraction.

### *Characterization of GS*

**Figure 1:**
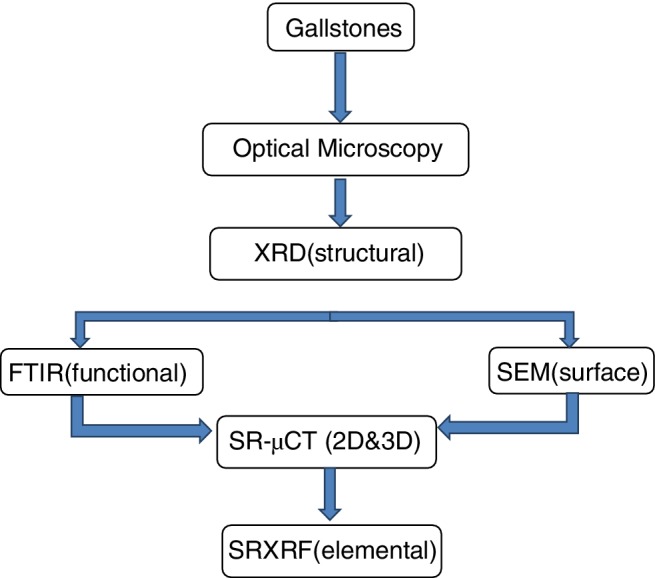
Sequential analysis of morphological and elemental analysis of gallstones. FTIR, Fourier transform infrared; SEM, scanning electron microscopy; SR‐μCT, synchrotron microtomography; SR‐XRF, synchrotron X‐ray fluorescence spectroscopy; XRD, X‐ray diffraction.

The gross morphological characterization of GS was carried out by optical microscopy, XRD, FTIR, and SEM (Table 1 and Fig. 1).

1. Optical stereo microscopy provides information on the gross appearance of the GS and is captured as an image.
2. XRD analysis utilizes PAN analytical Xpert‐pro powder X‐ray diffractometer. The crystalline and noncrystalline phases of the GS are depicted as peaks.^15^

Principles of XRD analysis: When an X‐ray of fixed wavelength and at a definite incident angle is bombarded on the crystal, reflected X‐rays are produced. For the waves to interfere constructively, a diffracted beam of X‐ray leaves the crystal at an angle equal to that of an incident beam, and this is represented as peaks.

As the next step, for the characterization of GS, FTIR and SEM analyses were carried out.

3 FTIR: The instrument Jasco Fourier Transform Infrared Spectrometer (Easton, Pennysylvania, United States America), Model FTIR‐6300, KBr mode in the 4000 to 400 per cm spectral domain, highlights the chemical functional groups of cholesterol and bilirubinate salts as peaks at the respective wavelengths.

Principles of FTIR analysis: The infrared spectroscopy depicts the interaction of sample with light radiation when electromagnetic waves interact with the polarity of the chemical bonds of the molecules. When there is no polarity in the molecule, the infrared interaction is inactive, and the molecule does not produce an infrared spectrum.

4 SEM using Carl Zeiss Thornwood, MA15/EVO 18 (New York, United States of America) with resolution of 3.0 nm at 30 kV provides information on the surface morphology of GS at the micro level and highlights the crystalline and noncrystalline nature of GS.
5 Synchrotron microtomography (SR‐μCT), a third‐generation synchrotron‐based X‐ray imaging beamline (Indus‐2, BL‐4),^16^ has a high sensitivity and fast image capacity, providing excellent insight into the initiation and promotion of the formation of GS, including mapping of the mineral deposition. For interpretation, the images are first reconstructed for computed 2D slice gray images and thereafter to 3D microtomographic images using the Octopus Imaging 3D tomography software (Gent, Belgium).
6 Synchrotron X‐ray fluorescence spectroscopy (SR‐XRF): This interprets the spectra using PyMca software; https://github.com/vasole/pymca. The elements are identified by the high resolution of brilliance and quantified from the graph.^17^

The study was approved by the institutional ethics committee in full compliance with acceptable international standards, including the Declaration of Helsinki.

## Results

**Figure 2:**
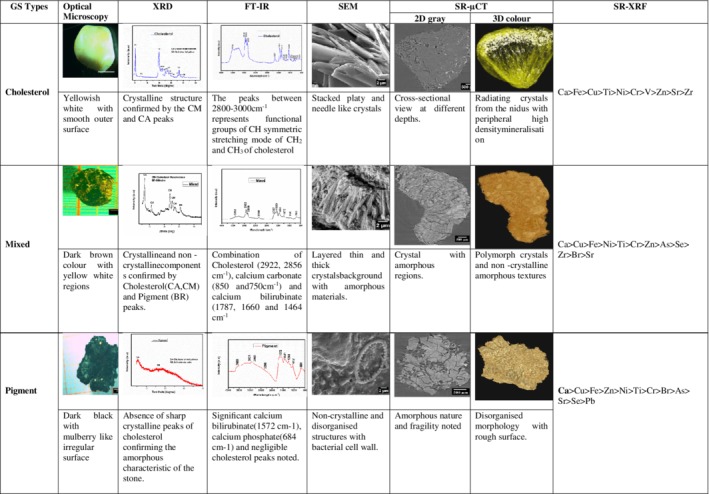
Prototype characteristics of cholesterol, mixed, and pigment gallstones (GS). BR, bilirubin; CA, cholesterol anhydrous; CM, cholesterol monohydrate; FTIR, Fourier transform infrared; SEM, scanning electron microscopy; SR‐μCT, synchrotron microtomography; SR‐XRF, synchrotron X‐ray fluorescence spectroscopy; XRD, X‐ray diffraction.

Figure 2 summarizes the morphological characteristics of prototype cholesterol, mixed, and pigment GS and Table 3 the elemental composition of the 10 GS.

**Table 3:** Elemental concentrations (ppm by weight) of gallstones measured by X‐ray fluorescence spectroscopy

Element	Samples									
	CH1	CH2	CH3	CH4	CH5	CH6	M1	M2	P1	P2
	Element concentration in ppm									
Ca	160.6	106.3	103.7	100.2	156.42	126.7	162.84	524.03	617.3	723.7
Ti	4.7	2.87	2.91	3.1	1.32	2.1	1.53	3.35	4.15	0.89
V	3.2	1.5	—	—	—	—		—	—	—
Cr	2.8	1.95	1.21	2.02	—	—	1.82	1.57	1.53	3.54
Mn	1.2	0.80	0.48	0.21	1.29	0.12	0.80	1.42	1.06	3.95
Fe	28.4	18.29	18.38	6.52	10.67	24.51	18.22	17.34	27.43	35.58
Ni	4.5	4.07	4.18	0.53	0.26	4.69	4.13	3.47	4.36	1.49
Cu	0.48	0.32	0.29	20.12	6.17	0.86	0.31	41.31	53.8	131.67
Zn	0.65	0.35	0.42	1.78	0.86	1.51	0.34	1.54	0.32	5.92
As	0.08	0.05	0.02	0.10	0.31	0.03	0.04	1.83	0.02	0.63
Se	—	—	—	—	0.04	—	0.01	0.76	—	0.13
Br	—	—	0.01	0.06	0.08	5.46	—	0.23	—	2.5
Sr	1.4	0.03	0.02	3.35	0.07	0.04	0.02	0.04	0.02	0.22
Zr	—	—	—	—	0.08	0.05	—	0.97	—	—
Pb	—	—	—	—	—	—	—	—	—	0.07

### *Cholesterol GS*

#### *Six representative samples were analyzed (CH1–CH6)*

**Figure 3:**
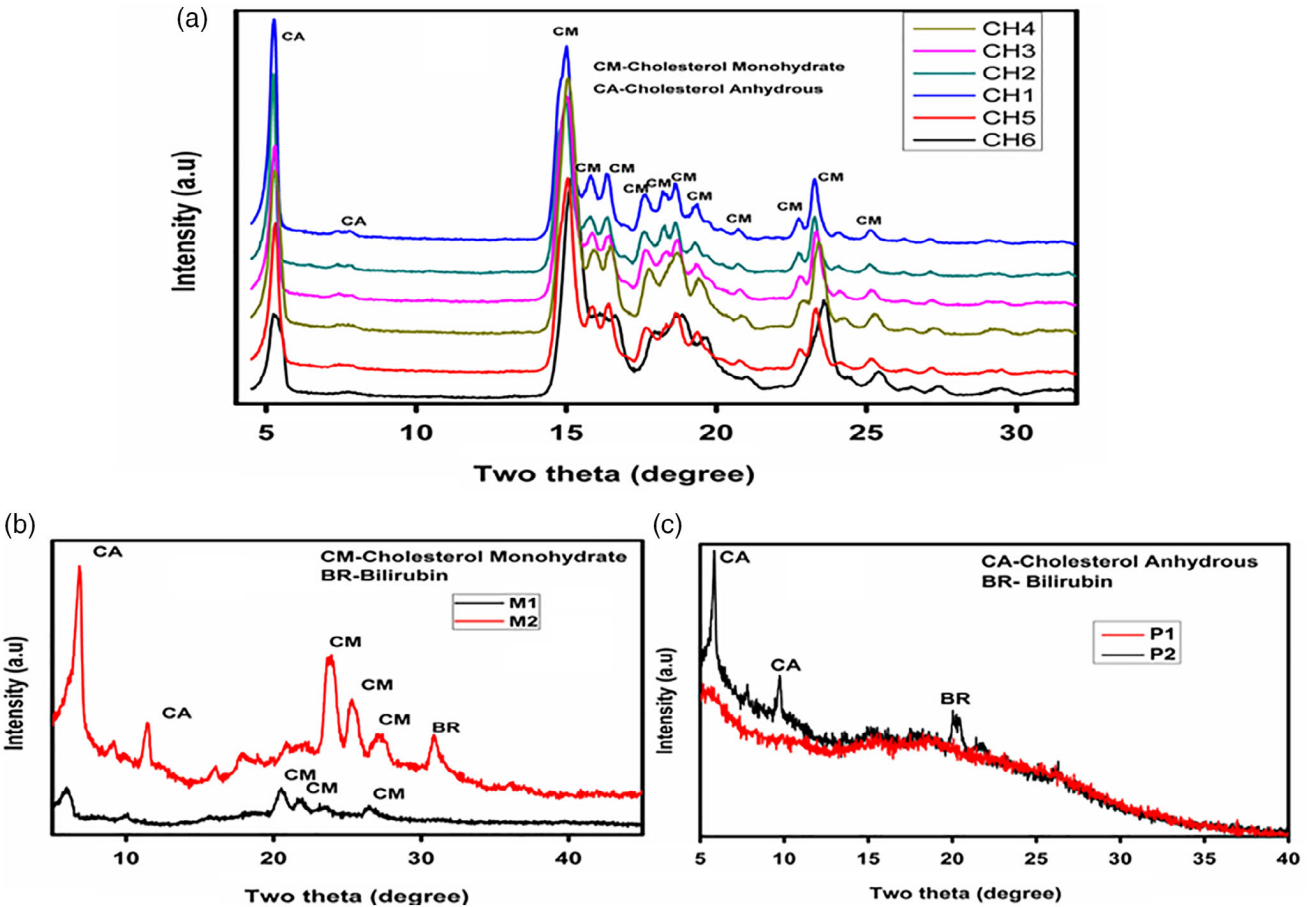
X‐ray diffraction patterns of (a) cholesterol, (b) mixed, and (c) pigment gallstones. (a): (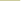), CH4; (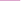), CH3; (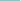), CH2; (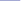), CH1; (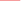), CH5; (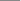), CH6. (b): (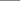), M1; (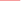), M2. (c): (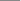), P1; (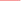), P2.

**Figure 4:**
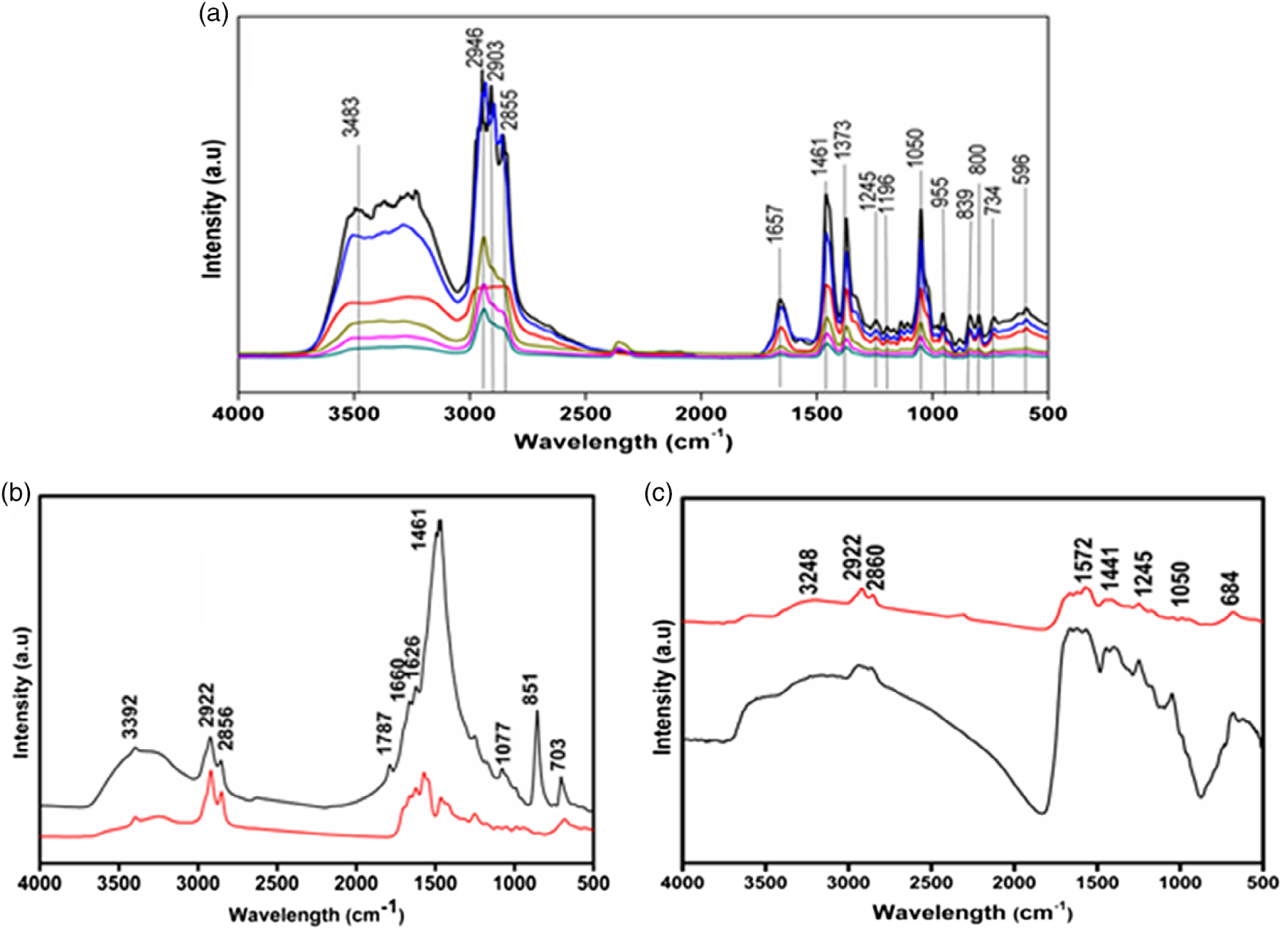
Fourier transform infrared spectrum of (a) cholesterol, (b) mixed, and (c) pigment gallstones. (a): (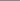), CH1; (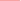), CH2; (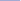), CH3; (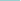), CH4; (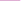), CH5; (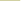), CH6. (b): (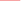), M2; (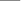), M1. (c): (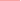), P2; (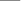), P1.

On optical microscopy, the cholesterol stones were yellowish‐brown in color with a smooth surface. XRD showed the characteristic peaks of cholesterol in both anhydrous and monohydrate forms. All the six stones, on FTIR (Fig. 4), showed broad bands in the wavelength range of 2500–3500 cm^−1^. The bilirubinate salts were seen between 1245 and 1657 cm^−1^. In addition, PO_4_^3−^ group and calcium carbonate were seen at 955 cm^−1^ and between 850 and 750 cm^−1^, respectively. SEM analysis uniformly showed a central core of orientated flakes of cholesterol crystals orderly stacked with thin needle‐like structures, which overlapped with the flakes. These were confirmed by XRD (Fig. 3) as cholesterol monohydrate and cholesterol anhydrous. The 2D slice gray‐scale image depicted the actual nucleation site with radiation toward the outer portion; 3D microtomography showed a high‐density mineral (white patches) in the periphery. On SR‐XRF, cholesterol GS had high amounts of calcium (average 160.6 ppm) and iron (average 28.4 ppm) (Table 3, Fig. 2).

### *Mixed GS (M1, M2)*

Grossly, these stones were dark brown with a well‐defined rough outer surface. On XRD and FTIR (Figs 3 and 4), one of the two mixed GS showed only cholesterol monohydrate, while the other showed peaks for both monohydrate and anhydrous forms of cholesterol. Both the GS showed the bilirubin in their respective wavelengths (1245 cm^−1^). SEM in one stone showed a layered formation of cholesterol and amorphous nature due to the free radical complex of bilirubin, while in the other, there was disorientation of small flakes with little fibrous material. The flakes were confirmed to be cholesterol monohydrate by XRD. The 2D slice image on (SR‐μCT) showed the polymorph nature of the crystals, with some discrepancy in their size and crystallographic arrangements. The smooth textures in some regions of the stone, as observed in the 3D images, corresponded to the bilirubinate salts and the organic substances. On SR‐XRF, the mixed GS had significantly more calcium (343.4 ppm *vs* 125.6 ppm) than the cholesterol GS, with almost similar concentrations of iron (17.78 ppm *vs* 17.6 ppm) (Table 3).

### *Pigment (P1, P2)*

Grossly, these were black, brittle, and amorphous with an irregular surface, that is, noncrystalline, with negligible amounts of the anhydrous form of cholesterol and bilirubin on XRD. Both the stones on FTIR analysis showed two peaks corresponding to calcium phosphate (684 cm^−1^) and bilirubinate salts (1572 cm^−1^) (Fig. 4). Noncrystalline and disordered arrangements due to the excessive layering of bilirubinate salts was confirmed by SEM, thereby confirming the amorphous nature of the pigment GS. This was further complemented by SR‐μCT. This was quite unlike the elemental constituents seen in the crystalline forms of cholesterol and mixed GS. On SR‐XRF, both the pigment GS had higher concentrations of calcium (670.5 ppm *vs* 343.4 *vs* 125.6), copper (92.73 *vs* 20.8 *vs* 4.69), and iron (31.50 *vs* 17.78 *vs* 17.6) compared to cholesterol and mixed GS (Fig. 2).

## Discussion

Our study has sequentially analyzed the morphological, chemical, and elemental constituents of prototype cholesterol, mixed, and pigment GS using various chemical and spectroscopic techniques. The optical stereo microscopy provided the gross appearance of the entire GS; XRD, the phase identification, that is, crystalline or noncrystalline; and FTIR and SEM, the functional groups. The latter not only confirmed the amorphous nature of GS but also demonstrated the presence or absence of bacteria and its degradation products. Finally, the identification of the probable initiator and promoter, that is, the nidus of GS, was possible with SR‐μCT using the 2D slice gray images and computed 3D microtomographic images. The concentrations of the minerals and elemental components that are likely to play a part in GS formation were quantified by SR‐XRF. Several other studies in literature similar to ours have provided some information on the chemical and spectroscopy techniques used by us, thereby providing some clues on the pathogenesis of GS.^18^, ^19^, ^20^ However, there are no studies from India addressing the role of SR‐μCT and SR‐XRF in mapping the distribution of the mineral and elemental components of GS.

It is hypothesized that the process of GS formation involves a complex process of precipitation of substances found in bile and includes cholesterol; calcium bilirubinate; and calcium salts of phosphate, carbonate, and palmitate. The role of high content of iron and copper in these stones in this process remains unknown. Do they constitute or are they involved in the nidus as in cholesterol GS, or are they simply secreted in bile as components in the mixture of organic material of GS? To this day, this remains unanswered. The composition of GS is eventually dependent on the composition of bile and the local epidemiological and possible genetic risk profile associated with it.

In our study, we hypothesize that the tightly stacked and arranged layered or cord‐like cholesterol is crystallized and precipitates around the nucleus as a twisting layer in supersaturated bile. The nidus/nucleus is likely to be the plate with needle‐like outward radiation of the crystals, with mineralization on its surface largely constituted by calcium and possible iron salts (Table 1). This was well demonstrated in our study with SR‐μCT.

Pigment GS are the dominant stones in the southern states of India. None of our patients with pigment GS in our earlier studies had hemolysis; bile was also less infective.^21^ Using various analytical instrumentation, we have confirmed the absence of a nidus in pigment GS and the presence of amorphous, nonfilamentous regions with significant amounts of calcium bilirubinate salts, iron, and copper. These elements seem to be intertwined with the amorphous material of the GS. Earlier, we reported high copper and iron content in pigment GS using PIXE analysis.^7^ Several other publications have reported that copper and iron play a major role in the formation of pigment GS.^22^ It is likely that the bilirubin derived from degradation of hemoglobin of a senescent red blood cell forms a complex compound derived from bilirubin and, together with copper and iron,^23^ represents a particulate generating the black stones. Furthermore, south Indian cuisine rich in *Tamarindus indica* is likely to contribute to the excess iron in our patients with pigment GS.^4^ One prototype pigment GS in our study had bacteria as documented by SEM (Fig. 2). Do the bacteria serve as a nidus for GS formation? This needs to be addressed through the analysis of more pigment GS.

Mixed GS have a combination of both cholesterol and calcium bilirubinate salts. The stones, through various analytical instrumentations in our study, showed both filamentous, platy crystals and amorphous areas in the GS, with excessive iron and calcium. Evidence from literature abounds with information on the pronucleating factors in bile, such as N‐aminopeptidase, fibronectin, immunoglobulins, acidic glycoproteins, mucin, and lipoproteins.^24^, ^25^ Cavalu *et al.*,^26^ using a deconvolution procedure, report that the random coils may represent the dominant secondary structure of proteins that may be involved in GS pathogenesis. The XRD pattern in their study confirmed that mixed and pigment GS had calcium bilirubinate complex formation, together with anhydrous and monohydrate forms of cholesterol. We also observed that both our pigment GS had calcium bilirubinate and small amounts of cholesterol. The form of cholesterol that precipitates in the nucleus and initiates GS formation is not yet known.

The elemental composition in our analyses of GS is similar to our earlier observations and is similar to reports from different geographical areas in Europe and Asia.^18^, ^19^, ^27^Calcium was the dominant element in all GS. High concentrations of iron and copper were found in pigment GS, similar to the reports from Nigeria^28^ and other studies from India,^18^ together with magnesium, iron, and copper. Cholesterol GS from Iran are rich in copper, similar to that seen in GS from India. Pigment GS across the board have uniformly shown high concentrations of iron and copper in different series. These include the composition reported from India (Uttarakhand) and Nigeria.^2^, ^4^, ^10^, ^11^, ^12^

In conclusion, analysis of the chemical composition using various spectroscopic techniques has provided some insight into the pathogenesis of GS. SEM provided information on the flake‐like and needle‐like crystals. SR‐μCT, in a 3D image, showed a radial layered arrangement of cholesterol crystals, with calcium salts in its periphery. The mixed GS showed a combination of high cholesterol and calcium bilirubinate salts by FTIR, with Sc‐microtomography showing polymorph crystals amongst the amorphous texture. Finally, pigment GS had high calcium bilirubinate on FTIR and an amorphous phase on XRD, with no pattern of morphology on SR‐μCT. Estimating the elemental concentrations of the three types of GS was possible with SR‐XRF, an instrument with a very high resolution compared to PIXE.

By understanding the GS morphology, its elemental constituents, and the composition of bile, in the future, it may be possible to crystallize a GS in vitro and also identify a solvent that can dissolve these GS.
